# Quantitative PCR primer design affects quantification of dsRNA‐mediated gene knockdown

**DOI:** 10.1002/ece3.5387

**Published:** 2019-06-23

**Authors:** Thomas Ogao Onchuru, Martin Kaltenpoth

**Affiliations:** ^1^ Department for Evolutionary Ecology, Institute of Organismic and Molecular Evolution Johannes Gutenberg University – Mainz Mainz Germany

**Keywords:** double‐stranded RNA, gene knockdown, Primer design, RNAi, RT‐qPCR

## Abstract

RNA interference (RNAi) is a powerful tool for studying functions of candidate genes in both model and nonmodel organisms and a promising technique for therapeutic applications. Successful application of this technique relies on the accuracy and reliability of methods used to quantify gene knockdown. With the limitation in the availability of antibodies for detecting proteins, quantitative PCR (qPCR) remains the preferred method for quantifying target gene knockdown after dsRNA treatment. We evaluated how qPCR primer binding site and target gene expression levels affect quantification of intact mRNA transcripts following dsRNA‐mediated RNAi. The use of primer pairs targeting the mRNA sequence within the dsRNA target region failed to reveal a significant decrease in target mRNA transcripts for genes with low expression levels, but not for a highly expressed gene. By contrast, significant knockdown was detected in all cases with primer pairs targeting the mRNA sequence extending beyond the dsRNA target region, regardless of the expression levels of the target gene. Our results suggest that at least for genes with low expression levels, quantifying the efficiency of dsRNA‐mediated RNAi with primers amplifying sequences completely contained in the dsRNA target region should be avoided due to the risk of false‐negative results. Instead, primer pairs extending beyond the dsRNA target region of the mRNA transcript sequences should be used for accurate and reliable quantification of silencing efficiency.

## 1 INTRODUCTION

The discovery of RNA interference (RNAi) revolutionized the study of gene functions in eukaryotes. This gene regulatory mechanism utilizes double‐stranded RNA (dsRNA) or short interfering RNA (siRNA) molecules to direct homologous‐dependent interference of gene activity (Novina & Sharp, 2004; Scott et al., 2013). The presence of dsRNA in eukaryotic cells triggers the RNAi machinery to initiate reactions leading to the methylation of histone proteins or destruction of mRNA transcripts resulting in transcription or translation inhibition, respectively (Novina & Sharp, 2004). This process begins with the cleavage of free dsRNA in the cytoplasm by RNaseIII endonuclease dicer into small interfering RNAs (siRNAs) that are picked up by the RNA induced silencing complex (RISC), a multi‐protein complex, which degrades the sense strands of the siRNAs and uses the antisense strands as guides for the destruction of target complementary mRNA transcripts before they are translated into proteins (Novina & Sharp, 2004; Scott et al., 2013). Alternatively, the antisense strands can recruit enzymes that methylate the histone proteins leading to the formation of a silenced chromatin, thereby inhibiting transcription (Novina & Sharp, 2004).

Naturally, RNAi regulates development and physiology, suppresses transposon activity, and provides defense against RNA virus infections in many organisms using endogenously expressed microRNAs or exogenously introduced viral dsRNA (Ambros, 2004; Obbard, Gordon, Buck, & Jiggins, 2009). However, this mechanism can also be exploited artificially to study functions of endogenous eukaryotic genes of interest through the introduction of synthetic dsRNA or siRNA molecules that trigger the host's natural RNAi machinery to silence the respective genes, which allows investigation into their specific functions. The artificial injection of dsRNA for a gene encoding the myofilament protein into the nematode *Caenorhabditis elegans* led to the discovery of dsRNA‐mediated RNAi silencing (Fire et al., 1998). Following its discovery, dsRNA‐mediated RNAi became a powerful research tool for understanding gene functions as well as a promising therapeutic candidate for the management of genetic disorders (Agrawal et al., 2003; Seyhan, 2011).

The success and extent of RNAi silencing differs between hosts, life stages, and genes of the same organism. This may be due to variability in the stability of dsRNA molecules in vivo, their uptake by target cells, and in vivo amplification and transmission of the silencing signal between cells to facilitate systemic or transgenerational silencing (Scott et al., 2013; Wang et al., 2016). In addition to these endogenous challenges, successful use or application of RNAi can be influenced by external technical factors, including the methods used for administering the dsRNA and measuring its success (Herbert, Coppieters, Lasham, Cao, & Reid, 2011; Holmes, Williams, Chapman, & Cross, 2010; Scott et al., 2013). It is therefore important that reliable and precise methods are used to evaluate efficacy and specificity of gene silencing following RNAi to avoid false‐positive or false‐negative results and consequently wrong conclusions (Herbert et al., 2011; Holmes et al., 2010). In addition to phenotypic observations, there are two standard methods for assessing the success of RNAi‐mediated gene silencing: real‐time quantitative PCR (RT‐qPCR) for quantifying depletion of relevant mRNA transcripts relative to controls, and western blotting or immunofluorescence for measuring the reduction in the amount of target proteins (Agrawal et al., 2003; Scott et al., 2013).

**Figure 1:**
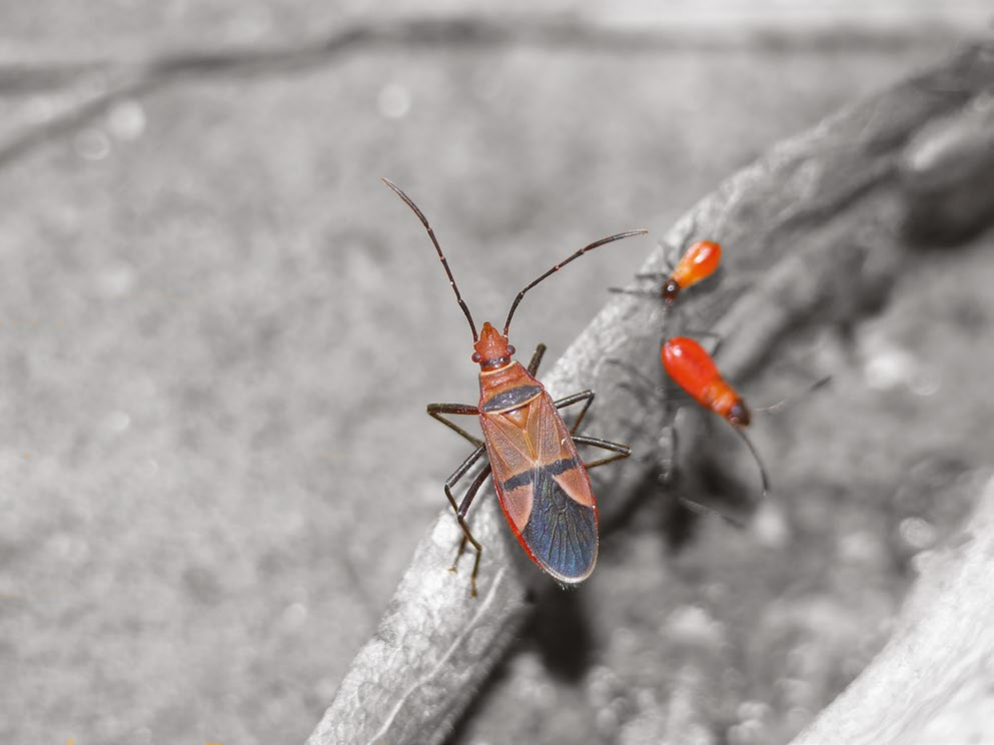
The African cotton stainer (*Dysdercus fasciatus*) adult insect (left) and its juveniles (right) maintain a symbiotic relationship with gut bacterial mutualists. These symbionts supplement the insect's cotton seed diet with limiting B‐vitamins and protect against trypanosomatid infections. Once established, the gut microbiota appears to be resistant to the host's immune effectors, as revealed by RNA interference of immune effector and upstream signaling genes. © Martin Kaltenpoth, Johannes Gutenberg University Mainz

While it is known that accurate quantification of target mRNA transcript levels by RT‐qPCR after gene knockdown with exogenous siRNAs depends on the selection of primer binding site relative to the siRNA cleavage site (Herbert et al., 2011; Holmes et al., 2010), it is unclear how RT‐qPCR primer selection affects quantification of dsRNA‐mediated RNAi gene knockdown, especially in relation to the expression levels of target genes. Our first insights into the importance of primer design in the accurate measurement of dsRNA gene silencing came about when we were studying the role of *Dysdercus fasciatus*’ (Hemiptera: Pyrrhocoridae; Figure 1) immune genes in the regulation of its gut bacterial symbionts (Onchuru & Kaltenpoth, 2019). Following dsRNA‐mediated RNAi, transcript levels of genes under low expression remained unchanged or were even higher in the treatment groups compared to the controls, while transcript levels of the highly expressed target genes decreased significantly as expected. To identify if this unexpected result was due to primer design and/or target gene expression levels, we designed two primer pairs for three genes with different levels of expression, respectively; one primer pair targeted the part of the mRNA transcript sequence that was entirely complementary to the dsRNA used for silencing and the other amplified a sequence extending beyond the region complementary to the dsRNA construct within the target mRNA. We report that the use of qPCR primers targeting a sequence that is completely contained within the dsRNA construct can lead to false negatives or an underestimation of gene knockdown in genes with low expression levels.

## 2 MATERIALS AND METHODS

**Figure 2:**
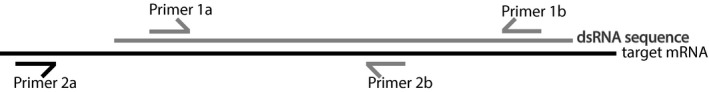
Schematic illustration of selected qPCR primer binding sites relative to the location of the dsRNA construct within the target mRNA. Primers were designed to amplify the mRNA sequence section that is complementary to the dsRNA used for silencing (Primer set 1) or amplify a sequence extending beyond the boundaries of the dsRNA construct within the target mRNA transcript (Primer set 2)

Total RNA extracted from *D. fasciatus* using the innuPREP RNA Mini Isolation Kit (Analytik Jena) was used for cDNA synthesis with Quantitect® Reverse Transcription kit (Qiagen) as per the manufacturer's guidelines. This cDNA was used as a template for the synthesis of dsRNA for *D. fasciatus*' antimicrobial peptide genes defensin 1 and defensin 2, which have low expression levels (<10 RPKM), and c‐type lysozyme gene with a higher expression level (>100 RPKM) using MEGAscript® RNAi kit (Thermo Fisher Scientific) following the manufacturer's protocol. The expression levels of the three genes are based on a previously published transcriptomic data which reported the normalized gene transcripts of the c‐type lysozyme to be at least 200 times higher than those of the different isoforms of the defensin genes (Bauer, Salem, Marz, Vogel, & Kaltenpoth, 2014). The synthesized dsRNA was used for in vivo RNAi gene knockdown that was performed by feeding 15 replicate individuals of second instar *D. fasciatus* nymphs that had been deprived of water for 24 hr with dsRNA for defensins (i.e., defensin 1 and defensin 2 in combination) or c‐type lysozyme, or nonsense dsRNA for GFP for the control group, respectively. In pilot experiments, we found that there was no difference in the degree of silencing when the two defensin genes are silenced independently or together. One week after dsRNA feeding, total RNA was extracted from one nymph per replicate treatment and used for cDNA synthesis as described above to measure gene knockdown success. Two qPCR primer sets (Table 1) were designed for each gene with primer BLAST using their respective sequences obtained from the *D. fasciatus* transcriptome (Bauer et al., 2014). One primer set amplified the section of the target mRNA sequence that was entirely complementary to the dsRNA sequence used for silencing, while the second primer set amplified the mRNA sequence extending beyond the region that was complementary to the dsRNA construct (Figure 2). Specificity of the primers was determined by blasting their sequences and those of their respective PCR products against a local BLAST database that was created using the *D. fasciatus* transcriptome. Blasting of the PCR product sequences also allowed us to exclude the possibility of off‐target gene silencing.

**Table 1:** qPCR primers used in measuring dsRNA‐mediated gene knockdown

Target gene	Primer Name	Product size (bp)	Primer sequence	Target site of RT‐qPCR primers	Pairing
Defensin1	Dfas_Def_1F	239	GTCCTTCTCCTGGTCTTCGC	inside dsRNA	Set 1
	Dfas_Def_1R		ACTGTCTTCTTGCAGCTCCC	inside dsRNA	
Defensin1	Def_for	273	CAACTTTCCAAACAAATCCACA	outside dsRNA	Set 2
	Dfas_Def_1R		ACTGTCTTCTTGCAGCTCCC	inside dsRNA	
Defensin2	Dfas_Def_2F	224	CTCGCACCTTCCTCCTTTGT	inside dsRNA	Set 1
	Dfas_Def_2R		CTATGGTCGCTGTCTCGGC	inside dsRNA	
Defensin2	Defensin‐1F	173	GGGTGTGAACCACTGGGATT	inside dsRNA	Set 2
	Defensin‐1R_Modified		TATGCGCCGCTATGGTC	outside dsRNA	
c‐type Lysozyme	Lyso_For_2	168	CCTCTGGCACTTGGTCTTCC	inside dsRNA	Set 1
	Lyso_Rev_2		AACAGCCACTACTGGTGCAA	inside dsRNA	
c‐type Lysozyme	Lyso_For_1	163	CTTTCCAACCCTGAATGCTC	outside dsRNA	Set 2
	Lyso_Reverse		AGCACGGACTACGGACTGTT	inside dsRNA	
18S rRNA	Firebug18S‐1F	198	CGGTGCTCTTTACCGAGTGT	Firebug 18S rRNA (Onchuru et al., 2018)	
	Firebug18S‐1R		AACGTCGCAATACGAATGCC		

Quantitative PCR was set‐up using these two primer sets to quantify gene knockdown success. The 10 µl qPCR reaction mixture contained 0.5 µl of each primer (10 µM), 5 µl SYBR‐mix, 3 µl of qPCR H_2_O, and 1 µl of either the cDNA template or a standard or a negative control (H_2_O). The reaction mixture was run on the Rotor‐Gene Q cycler (Qiagen, Hilden, Germany) with the following cycling conditions: 95°C initial denaturation for 5 min followed by 95°C denaturation for 10 s, touchdown annealing for 15 s at 68–60°C for the first eight cycles, then 60°C annealing for the remaining 37 cycles for 15 s, extension of 72°C for 10 s, and a final melting curve analysis from 65°C to 99°C with a temperature raise of 1°C for each step. Quantification of each target gene was performed with the Rotor‐Gene Q software as described by (Onchuru, Martinez, & Kaltenpoth, 2018) using external standard curves amplified with similar conditions from serial dilutions (10^10^–10^2^ copies/µl) of purified PCR product of the respective gene. The transcript copy numbers of each gene were normalized with the number of host 18S rRNA transcripts that were quantified by qPCR using previously described primers (Table 1; Onchuru et al., 2018) and conditions described above. Differences in the normalized transcript copy numbers between treatments and controls were evaluated using Wilcoxon‐signed rank tests and plotted using boxplots as implemented in SPSS Version 23 (IBM). Part of the data analyzed here, that is, gene expression using primers targeting mRNA sequence extending beyond the dsRNA target site, is published in (Onchuru & Kaltenpoth, 2019).

## 3 RESULTS

**Figure 3:**
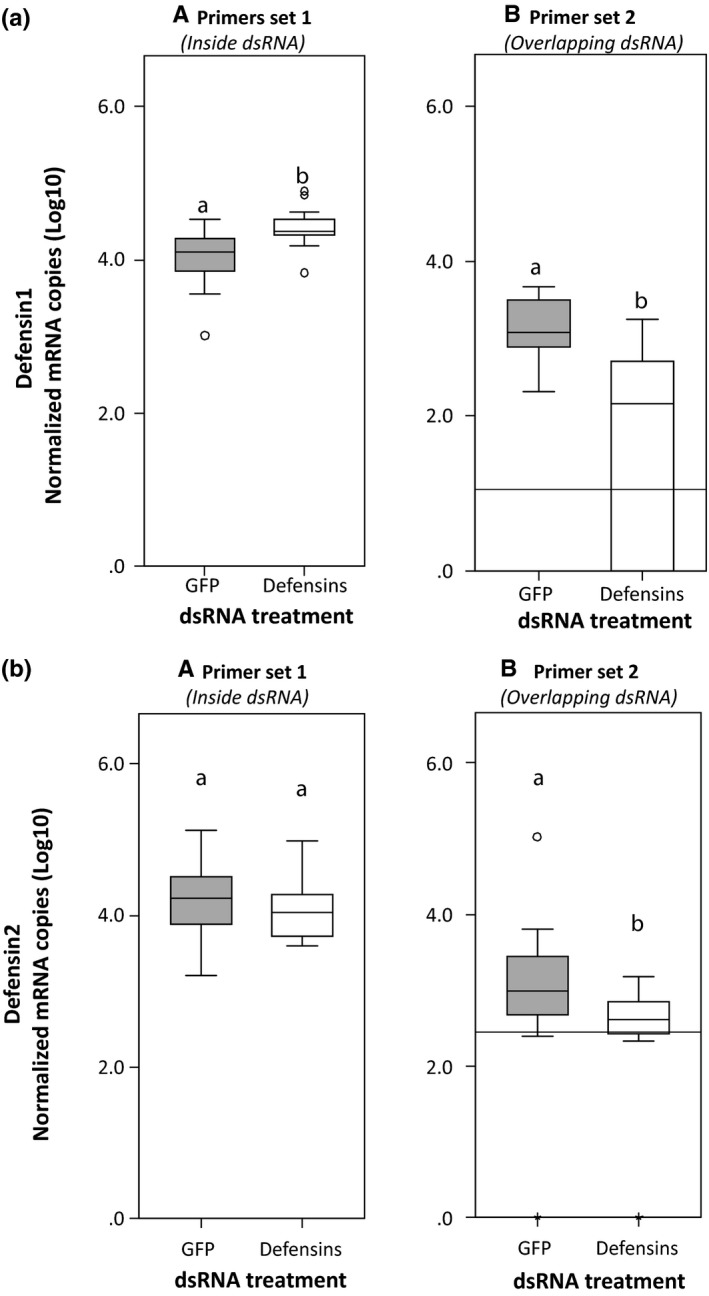
Quantification of transcript levels of (a) defensin 1 and (b) defensin 2 genes with low expression levels using two different primer sets. (A) Quantification with primers binding and amplifying the mRNA transcript sequence that is complementary to the dsRNA sequence used for silencing, and (B) quantification with primers binding and amplifying a sequence of the target mRNA transcript extending beyond the dsRNA construct. Significant differences are indicated by different letters above the boxes. Boxes comprise 25–75 percentiles, lines in boxes represent medians, whiskers denote the range, and circles represent outliers. Detection threshold is 0 if not indicated by the gray horizontal line (negative control in the qPCR)

**Figure 4:**
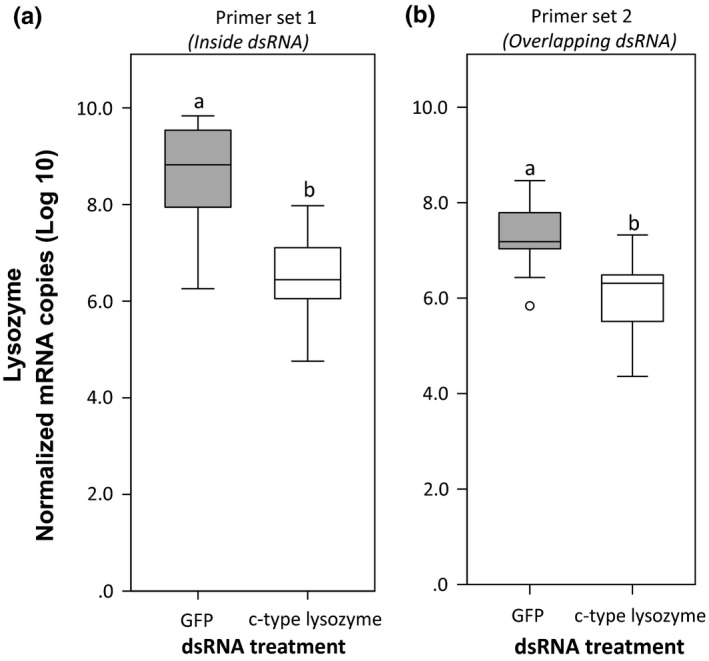
Quantification of transcript levels of the highly expressed c‐type lysozyme gene using two different primer sets. (a) Quantification with primers binding and amplifying the mRNA transcript sequence that is complementary to the dsRNA sequence used for silencing, and (b) quantification with primers binding and amplifying a sequence of the target mRNA transcript extending beyond the dsRNA construct. Significant differences are indicated by different letters above the boxes. Boxes comprise 25–75 percentiles, lines in boxes represent medians, whiskers denote the range, and circles represent outliers

We compared the effect of qPCR primer binding site within the mRNA transcript sequence on the accurate quantification of gene knockdown following dsRNA‐mediated RNAi for three genes with different expression levels. Quantifying transcript levels of target genes with primers targeting the mRNA sequence that is complementary to the dsRNA used for gene silencing (primer set 1, Figure 2) indicated a significant increase or an insignificant decrease of transcript levels in treated animals compared to the controls for genes with low expression levels (Figure 3), but not for a highly expressed gene (Figure 4). With this primer pair, expression of defensin 1 gene increased significantly by 119% in the knockdown group compared to the controls (Figure 3a, Wilcoxon‐signed rank test, *Z* = −2.953, *p* = 0.003) while the expression of defensin 2 gene in the knockdown group decreased insignificantly by 25% (Figure 3b, Wilcoxon‐signed rank test, *Z* = −0.966, *p* = 0.334). However, for the highly expressed c‐type lysozyme gene, a significant knockdown of up to 99% was observed with this primer set (Figure 4, Wilcoxon‐signed rank test, *Z* = −3.237, *p* = 0.001). On the other hand, measuring gene knockdown with primers targeting the sequence extending beyond the dsRNA construct within the target mRNA transcript (primer set 2, Figure 2) revealed a significant gene knockdown for all three genes, regardless of the gene's expression levels. With this primer set, a decrease of 93%, 84%, and 94% was recorded for c‐type lysozyme, defensin 1, and defensin 2 genes, respectively, in the knockdown group compared to the controls (Figures 3 and 4, Wilcoxon‐signed rank test, *Z* = −3.067, *Z* = −2.953, and *Z* = −2.329, respectively, *p* < 0.05).

## 4 DISCUSSION

Interfering with the activity of genes through RNAi‐mediated gene silencing and evaluating the host phenotypic changes is a powerful approach to determine specific gene functions. To confirm that the observed phenotypic changes are due to the knockdown of target genes and not off‐target effects, the concentrations of residual target gene transcripts and proteins are measured with RT‐qPCR and western blotting, respectively. In this study, we sought to understand the importance of qPCR primer design and host gene expression levels on the quantification of residual mRNA transcripts following dsRNA‐mediated RNAi. Our findings suggest that the target gene expression levels and the choice of primer binding site relative to the mRNA sequence targeted for silencing are important factors to consider when designing qPCR primers for evaluating RNAi success. Specifically, for genes with low expression patterns, quantification of mRNA transcripts with primers targeting an amplicon that is contained within the dsRNA target region resulted in an underestimation of the degree of silencing or in false‐negative results. However, this problem could be circumvented by using primers targeting an amplicon extending beyond the dsRNA target region within the mRNA transcript.

Efficacy and duration of RNAi‐mediated gene silencing varies not only between organisms but also between genes of the same organism (Scott et al., 2013; Wang et al., 2016). This disparity may be explained by variation in the RNAi machinery or extracellular enzymatic capacity of different organisms or tissues to degrade exogenous dsRNA molecules, which affects their stability in vivo and consequently silencing efficiency (Scott et al., 2013; Spit et al., 2017; Wang et al., 2016). Additionally, in some organisms, the silencing signal can be amplified by different mechanisms, for example, the RNA‐dependent RNA polymerase (RdRp), which uses siRNAs generated from the diced primary dsRNA molecule as primers to copy the target mRNA resulting in the formation of secondary dsRNA (Sijen et al., 2001). Our findings suggest that variation of RNAi between genes may be as a result of quantification errors influenced by improperly designed RT‐qPCR primers and differences in gene expression. In *D. fasciatus* insect, a significant reduction of the target mRNA transcripts is achieved for up to 2 weeks following dsRNA‐mediated RNAi (Onchuru & Kaltenpoth, 2019), a clear indication that either the dsRNA remains stable in vivo or the silencing signal is amplified resulting in sustained knockdown for this duration. The presence of residual primary or in vivo amplified dsRNA molecules can influence quantification of gene knockdown during qPCR. Stable dsRNA molecules may be extracted during total RNA extraction, reverse transcribed and quantified during RT‐qPCR. This results in an overestimation of the gene expression levels in the target knockdown treatments as compared to controls leading to an underestimation of gene knockdown or complete false negatives.

Our results corroborate the findings of other studies showing the importance of primer design in the quantification of intact mRNA transcripts after RNAi. In siRNA‐mediated RNAi experiments, the choice of RT‐qPCR primer binding position relative to the siRNA‐mediated cleavage site has an effect on the quantification of target mRNA transcripts (Herbert et al., 2011; Holmes et al., 2010). For example, after siRNA‐mediated cleavage, degradation of the generated 3’ mRNA fragment may be blocked; hence the use of primers targeting this fragment leads to an underestimation of the degree of RNAi‐mediated gene silencing (Holmes et al., 2010; Mainland, Lyons, Ruth, & Kramer, 2017). On the other hand, using primers too close to the siRNA cleavage site results in false‐positive results when contaminating siRNA molecules extracted with total RNA inhibit RT‐qPCR (Herbert et al., 2011).

RNAi is a useful tool for research and therapeutics (Agrawal et al., 2003; Seyhan, 2011). To successfully exploit this technique, however, a number of considerations must be taken into account when designing dsRNA‐mediated RNAi experiments. Besides optimizing dsRNA dosage, delivery, and cellular uptake with cell membrane penetrating peptides and increasing stability of dsRNA in the extracellular environment by knocking down dsRNases to improve RNAi efficiency (Spit et al., 2017), accurate quantification of successful RNAi gene silencing is key to avoiding incorrect conclusions. With RT‐qPCR being the most common and widely used method for the quantification of intact target mRNA transcripts after silencing, its reliability is essential regardless of the gene or organism being studied. Based on our findings, we recommend the use of primers amplifying the mRNA transcript sequence extending beyond the dsRNA target region of the mRNA transcript to ensure accurate quantification of RNAi gene knockdown, especially in genes with low expression patterns.

## CONFLICT OF INTEREST

None declared.

## AUTHOR CONTRIBUTIONS

M.K. and T.O.O. designed the study. T.O.O. performed the experiments and analyzed the data. T.O.O. wrote the manuscript, which M.K. reviewed and critically commented on. Both authors approved and are accountable for the final version for publication.

## Data Availability

The gene expression raw data measured by qPCR are available from the Dryad repository (https://doi.org/10.5061/dryad.28n8d6t).
